# Exosomes derived from cardiac progenitor cells attenuate CVB3-induced apoptosis via abrogating the proliferation of CVB3 and modulating the mTOR signaling pathways

**DOI:** 10.1038/s41419-019-1910-9

**Published:** 2019-09-18

**Authors:** Xin Li, Zuocheng Yang, Wenyuan Nie, Jie Jiang, Shentang Li, Zhuoying Li, Lang Tian, Xing Ma

**Affiliations:** 1 Department of Pediatrics, the Third Xiangya Hospital, Central South University, Changsha, China; 2 Department of Urology, Chinese People’s Liberation Army, 89th Hospital, Weifang, Shandong China; 3 Sate Key Laboratory of Advanced Welding and Joining, Harbin Institute of Technology (Shenzhen), Shenzhen, China

**Keywords:** Stem cells, Cardiomyopathies

## Abstract

Viral myocarditis is potentially fatal and lacking a specific treatment. Exosomes secreted by cardiac progenitor cells (CPCs) have emerged as a promising tool for cardioprotection and repair. In this study, we investigated whether CPCs-derived exosomes (CPCs-Ex) could utilize the mTOR signal pathway to reduce the apoptosis in viral myocarditis. In vitro, exosomes were, respectively, added to H9C2 cells after CVB3 infection to detect the anti-apoptosis effect of CPCs-Ex. Compared with the controls, the apoptosis rate was reduced, accompanied with the depressed expression of viral capsid protein 1 (VP1) and pro-apoptosis factors of Bim/caspase families. Meanwhile, the phosphorylation of Akt, mTOR, and p70S6K were promoted, but that of 4EBP1 was suppressed. In vivo, the results of apoptosis, expression of CVB3 and pro-apoptosis factors, and phosphorylation of Akt/mTOR factors of CVB3-infected cardiomyocytes were consistent with that of vitro. Following that, we use Rapamycin and MK-2206 to inhibit the Akt/mTOR signaling pathway, meanwhile, Rattus 4EBP1, p70S6K, Akt1 and Akt2 were transfected to H9C2 cells to establish the stably transfected cell lines. In the group with Rapamycin or MK-2206 pretreatment, CPCs-Ex also could decrease the apoptosis of H9C2 cells and expression of CVB3 mRNA, followed by decreased expression of apoptosis factors. In Akt2, p70S6K and 4EBP1 overexpression groups, CPCs-Ex promoted CVB3-induced apoptosis, VP1 expression and cleavage of caspase-3. Our results therefore identify CPCs-Ex exerts an anti-apoptosis effect in CVB3-infected cells by abrogating the proliferation of CVB3 and modulating the mTOR signaling pathways as well as the expression of Bcl-2 and caspase families. Viral myocarditis, mainly caused by CVB3 infection, is lacking a specific treatment. Our study identified an anti-apoptosis role of CPCs-Ex in CVB3-infected cells and rats, which shown that CPCs-Ex may be an effective tool to treat viral myocarditis. We believe that with more in-depth research on the functionality of CPCs-Ex, there will be a breakthrough in the treatment of viral myocarditis.

## Introduction

Viral myocarditis (VMC) is a common cause of dilated cardiomyopathy and sudden cardiac death^1^. Therefore, finding effective therapies for VMC is still a big challenge, and recent researches on cardiac progenitor cells (CPCs) may provide a turning point for the treatment of VMC^2^.

CPCs are a group of heterogeneous cells distributed throughout the heart and able to differentiate into several cell types, such as cardiomyocytes (CMs), vascular smooth muscle cells and endothelial cells (ECs), holding great promise for cardiac regeneration and functional reconstruction. After years of investigation, stem-cell therapy has yielded encouraging results in heart repair^3–5^. Moreover, CPCs from adult heart have emerged as better candidates for cardiac cell therapy compared with stem cells from bone marrow or adipose tissue^6^. Nevertheless, direct transdifferentiating into cardiac tissues is considered unlikely. The mechanism of adult stem-cell therapy has been tested to be mediated through paracrine release of extracellular vesicles containing growth factors and cytokines to exert anti-apoptosis, suppress immunity, and promote angiogenesis^6,7^. And exosomes may be the major active components of extracellular vesicles derived from CPCs^8^.

Exosomes are nano-sized (30–100 nm) particles secreted via exocytosis from dendritic cells (DCs), macrophages, T cells and cells of other tissue origins under physiological and pathological conditions, carrying proteins, lipids, and regulatory nucleic acids such as small and large non-coding RNAs into recipient cells^9^. Several pre-clinnical studies have suggested a better therapeutic role of CPCs-derived exosomes (CPCs-Ex) for cardiac repair compared with cell transplantation^10^. Indeed, CPCs-Ex containing specific agents have been effectively used to treat cardio-vascular disease. Chen et al.^11^ reported that Sca1 + CPCs-Ex can reduce cell apoptosis via inhibiting caspase-3/7 activation in a mouse model of acute myocardial ischemia/reperfusion. CPCs-Ex enriched with miR-21 were reported to prevent cardiomyocytes apoptosis by targeting PDCD4^12^. Recently, a deep proteomic analysis of neonatal CPCs-Ex conducted by Sudhish Sharma et al.^13^ identified a high level of insulin-like growth factor-1 (known to promote cardioprotection, inhibit apoptosis), suggesting an anti-apoptosis role of CPCs-Ex.

Coxsackievirus B (CVB), a member of the *Picornaviridae* family, is a common enterovirus that can cause various human systemic inflammatory disease such as myocarditis, meningitis, and pancreatitis and the six CVB serotypes are each responsible for different diseases and syptomes^14^, of which the infants and children are more susceptible^15–17^.

VMC still lacks effective treatments in this aria. Several preclinical stem-cell therapies have made some progress in reducing inflammation and improving myocardial function, but they are still not satisfactory^4,18,19^. As a cell-free therapeutic method, exosomes could avoid many of the limits of cell therapy. Barile et al.^20^ has demonstrated that CPCs-Ex could prevent staurosporine-induced cardiomyocyte apoptosis and they were more cardioprotective than MSCs-secreted exosomes. But the role of CPCs-Ex in VMC was still unexplored. Here we investigated the cardioprotective effect of the CPCs-Ex for myocardial cells in CVB3-induced myocarditis model, which is mainly through abrogating the CVB3 proliferation as well as regulating the expressions of mTOR signaling pathway and Bcl-2, caspase families. The fruitful work offers a possible cell therapy approach for viral myocarditis diseases.

## Materials and methods

### Cell isolation and culture

Cardiac progenitor cells (CPCs) were generated from the hearts of 2-month-old male Sprague-Dawley (SD) rats following these steps. Briefly, the first, the rat heart tissue was aseptically isolated and chopped with scissors and scalpel (finer as possible), and the tissue debris were loaded into 15 ml tube. Second, 5 ml type IV collagenase digestion (1 mg/mL, containing Dnase I) was added, digested 5 mins in 37 °C, three times in total. After that, discarding the supernatant by standing or briefly centrifuged. Then, after cleaned with PBS, the tissue block was re-suspended with CEM medium and noculated in 20 µg/mL FN coated Petri dish. After 14 days, the dishes were gently washed with PBS and then digested for 1–2 min with a 0.05% trypsin (preheated at 37 °C). The collected cells were cultured and maintained in complete media containing M199 (Corning, Corning, NY, USA), EGM-2 (Lonza, Walkersville, MD, USA), 10% exosomes-depleted FBS, 10 nM b-FGF, 1% MEM nonessential amino acids (Gibco, USA), and penicillin–streptomycin (Gibco, USA).

### Exosome isolation and purification

The CPCs-Ex isolation and purification were followed by the procedure of ExoQuick-TC^TM^ Exosome Isolation Reagent (System Biosciences, USA). When exosomes were prepared from media, the media was first concentrated from 50 mL to 130 µL with Amicon Ultra filter (Millipore, Billerica, MA) with a 100000-molecular weight cutoff before ExoQuick treatment^21,22^.

### Transmission electron microscopy

As Hinescu et al.^23^ described before, exosome pellet was re-suspended and fixed with 2.5% glutaraldehyde, post-fixed in buffered 1% OsO_4_ with 1.5% K_4_Fe(CN)_6_, embedded in 1% agar, and processed according to the standard Epon812 embedding procedure. The presence and the size of exosomes were determined using transmission electron microscopy (TEM, FEI Company, Netherlands) at 80 kV. Micrographs were used to measure the diameter of exosomes.

### Exosome labeling with DioC18(3) and uptake study

To assess in vitro uptake of CPCs-Ex by H9C2 cells, the purified CPCs-Ex were labeled with DioC18(3) (DiO perchlorate, Dio) green fluorescent labeling kit (Yeasen Company, China) according to the procedure. The Dio concentration is 0.5 µM per microliter exosomes from 1 × 10^4^ cells. The labeled exosomes were stained with Dio dye in 100 µL DMSO diluted by DMEM for 20 min at 37 °C, and another equal volume of serum without exosomes was added to stop the labeling. Then, the labeled CPCs-Ex were incubated with H9C2 cells for 12 h at 37 °C and determined with the fluorescence microscope.

### Animal experiments

All rats (male SD rats, 0–3 days after birth) used in our study were obtain form the department of Laboratory Animals, Central South University, China. And all experimental protocols were approved by the Institutional Review Board (IRB) of Third Xiangya Hospital, Central South University, China. All animal procedures conformed to the guidelines from Directive 2010/63/EU of the European Parliament on the protection of animals used for scientific purposes. In CVB3 group (*n* = 12), rats were injected CVB3 with 10^4^ TCID50 enterocoelialy to establish the viral myocarditis modal. In addition, the rats infected with CVB3 were injected with CPCs-Ex intravenously at 24 h p.i. in CPCs-Ex group (*n* = 12). Afterward, all rats were sacrificed by cervical dislocation at 7 days p.i. and acquired the heart tissue. Then, the tissue structural modification was observed using hematoxylin-eosin (HE) staining and TEM. The apoptosis of myocardial cells was detected by TdT-mediated dUTP nick-end labeling (TUNEL) as well as the immunofluorescence (IF). Real-time fluorescence quantitative PCR and western blot analysis were used to examine the expression of mTOR signaling factors and apoptosis proteins.

### Plasmid construction and cells stable transfection

The empty vector (pcDNA3.1-myc-HisA(-)) were the generous gifts from Dr. Qiaojia Huang (Molecular Medicine Research Center of Fuzhou General Hospital, Nanjing Military Region) preserved in our library. The sequences of Rattus 4EBP1, p70S6K, Akt1, and Akt2 were acquired from NCBI and amplified from cDNA of H9C2 cell line. The pcDNA3.1-myc-HisA(-)-4EBP1, pcDNA3.1-myc-HisA(-)-p70S6K, pcDNA3.1-myc-HisA(-)-Akt1, and pcDNA3.1-myc-HisA(-)-Akt2 constructs expressing Rattus 4EBP1, p70S6K, Akt1, and Akt2, respectively, were expressed and extracted from *Escherichia coli*. After that, H9C2 cells were obtained from the Institute of Oncology, Central South University and grown in Dulbecco’s modified Eagle’s medium (DMEM, Gibco, Life Technologies, Inc.) containing 10% heat-inactivated fetal bovine serum (FBS, Gibco, Life Technologies, Inc.) which was extracted exosome by ultracentrifuging at 37 °C in a humidified incubator with 5% CO_2_. H9C2 cells at 60% confluence were transfected with pcDNA3.1-myc-HisA(-)-4EBP1/p70S6K/Akt1/Akt2 or an empty vector, respectively. For stable transfection of H9C2 cells, 4 μg of expression plasmid was introduced by using Lipofectamine 2000 reagent (Invitrogen, Life Technologies) according to the manufacturer’s instructions. At 5–6 h post transfection, the cells were refreshed by the DMEM containing 10% exosome-free FBS. And then, after 12–24 h, Geneticin (G418) (Gibco, Life Technologies, Inc.) was added as a selective marker at the final concentration of 800 μg mL^−1^ for selecting the transfected clones and at the final concentration of 400 μg/mL for the maintenance of transfection during the course of experiments^24^.

### Virus infection

Coxsackievirus B3 (CVB3) Nancy strain was obtained from Shanghai Jiao Tong University School of Medicine, propagated in H9C2 cells and stored at −80 °C in our laboratory. The titer of virus was examined prior to each experiment. Cells in CVB3 groups were infected at 100 TCID50 with CVB3 or with DMEM containing 2% exosome-free FBS for Sham. After 1 h infection, cells were washed with phosphate-buffered saline (PBS) and replenished with fresh DMEM containing 2% exosome-free FBS, kept growing in a humidified incubator with 5% CO_2_.

### Cell apoptosis analysis

Apoptosis in different groups was determined by fluorescence-activated cell sorting (FACS) analysis of cells stained with Annexin-V FITC and propidium iodide (PI, Promega) by flow cytometer (FCM). At 12 and 24 h p.i., cells were harvested using 0.25% EDTA-Trypsin (Gibco, Life Technologies, Inc.). After centrifugation, cell pellets were washed twice with cold PBS, and then the cells pellets were incubated with Annexin-V FITC and propidium iodide to achieved double staining, according to the manufacturer’s instructions. The mixture was incubated in the dark for 15 min at room temperature. Afterward, 400 μL of 1 × binding buffer was added to each tube and cells were immediately analyzed by FACS Calibur flow cytometry (Becton Dickinson, USA)^24^.

### Real-time fluorescence quantitative PCR

H9C2 cells in different groups were harvested at 12 and 24 h p.i. First, mRNA was extracted following the method of RNA Extracted Kit (Omega, USA). Next, mRNA was reverse transcribed into cDNA following the RevertAidTM First Strand cDNA Synthesis Kit (Thermo, USA). At last, real-time fluorescence quantitative PCR (RT-PCR) amplification was done following the SYBGREEN PCR Master Mix Kit (ABI, USA). The primer sequences are on the table below (Table 1). The amplification profile was 10 min at 95 °C, 15 s at 95 °C, 30 s at 60 °C for 40 cycles. Signal of a gene was normalized with β-actin using the formula ΔCT = CT target − CT reference. And ΔΔCT = mean value of ΔCT control − ΔCT sample. At last, 2-ΔΔCt method was used to calculate the differences of mRNA transcript level^21^.

**Table 1:** Primer sequences

β-actin-F	AAGATCAAGATCATTGCTCCTCC
β-actin-R	TAACAGTCCGCCTAGAAGCA
CVB3-F	TGGTGGGCTATGGAGTATGG
CVB3-R	CACTGGATGGGGTGTTGTCT
mTOR-F	CCTCGGCACATCACTCCCTT
mTOR-R	GCTCCTACATTTCAGCACCCACT
AKT1-F	TACCTGAAGCTACTGGGCAAGGG
AKT1-R	CGGTCGTGGGTCTGGAATGAG
AKT2-F	GATGGTAGCCAACAGTCTGAAGCA
AKT2-R	CCCTTGCCGAGGAGTTTGAGATA
BCL-2-F	GGGGAGCGTCAACAGGGAGA
BCL-2-R	GAGACAGCCAGGAGAAATCAAACAGA
Bax-F	TTTGCTACAGGGTTTCATCCAGG
Bax-R	CAGCTCCATGTTGTTGTCCAGTTC
Bim-F	GAGTTCAATGAGACTTACACGAGGAG
Bim-R	CAGACGGAAGATGAATCGTAACAG
Caspase-9-F	CGGTGGACATTGGTTCTGGC
Caspase-9-R	AGTTCACGTTGTTGATGATGAGGC
Caspase-3-F	TGGAACGAACGGACCTGTGG
Caspase-3-R	CGGGTGCGGTAGAGTAAGCA
4EBP1-F	CAAAGGACCTGCCAACCATTCCA
4EBP1-R	TTCACCACCTGCCCGCTTATCTT
p70S6K-F	TATTGGTAGCCCACGAACGC
p70S6K-R	CCACTTGTTTCCATTGGGTATT

### Western blot analysis

CPCs-Ex, rat lymphocytes and H9C2 cells in different groups were washed twice with ice-cold PBS and then kept on ice for 10 min. In short, 80 μL lysis buffer (Beyotime Institute of Biotechnology) containing 0.1% phenylmethylsulfony (PMSF, Cwbio, China) was added in each well. Afterward, cell lysates were collected, and the precipitate was discarded after centrifugation. Then 30 μg extracted protein were fractionated on sodium dodecyl sulfate—10 to 12% polyacrylamide gels, electrophoretically transferred to 0.45 μm PVDF membranes (Millipore Corporation), and blocked with PBS containing 0.5‰ Triton-100 (Cwbio, China) and 5% nonfat dry milk for 1 h. Afterward, the membrane was incubated with the primary antibody (monoclonal anti-CD63 (Abcam, USA)), monoclonal anti-CD-81 (Santa Cruz Biotechnology, Inc., USA), monoclonal anti-4EBP1 antibody (Cell Signaling Technology), monoclonal anti-p70S6K antibody (Cell Signaling Technology), monoclonal anti-enterovirus antibody (Dako Co.), monoclonal anti-bim antibody (Cell Signaling Technology), polyclonal anti-bax antibodies (Cell Signaling Technology), monoclonal anti-caspase-9 antibody (Cell Signaling Technology), polyclonal anti-caspase-3 antibodies (Cell Signaling Technology), ponoclonal anti-Akt antibody (Cell Signaling Technology, USA), monoclonal anti-pAkt antibody (Cell Signaling Technology, USA), monoclonal anti-mTOR antibody (Cell Signaling Technology, USA), monoclonal anti-pmTOR antibody (Cell Signaling Technology, USA), monoclonal anti-BNP antibody (ab19645), monoclonal anti-c-Kit antibody (Biotin, ab25022), ponoclonal anti-CK-MB antibody (Proteintech: 15137-1-AP), ponoclonal anti-cTnI antibody (Proteintech: 21652-1-AP) and monoclonal anti-β-actin antibody (Proteintech Group, Inc., USA) at 4 °C overnight, followed by incubation with horseradish peroxidase-conjugated secondary antibodies (Proteintech Group, Inc., USA). At last, protein expression was detected by enhanced chemiluminescence (Pierce Professional Resources, USA).

### Immunofluorescence

Heart tissue was washed thrice with cold PBS at each time point, fixed in cold paraformldehyde for 30 min, and then blocked with PBS containing 5% Tween (Cwbio, China) and 3% bovine serum albumin (BSA) for 2 h at 4 °C, then incubated with monoclonal anti-enterovirus antibody (Dako Co.) for 4 h at room temperature. Followed by fluorophore-labeled donkey anti-mouse IgG (H + L) antibody (Invitrogen, Life Technologies), DAPI (Roche Group, Switzerland) was incubated for 3 min at room temperature to dye the nucleus at last and observed under Olympus microscope (Olympus Corporation, Japan) equipped with a Metamorph image acquisition system (DP2-BSW software).

### Statistical analysis

Two-way analysis of variance with multiple comparisons and paired Student’s *t*-tests were performed. Data were presented as the mean ± standard error (S.E.). The *P* value of <0.05 was considered significant.

## Results

### CPCs-Ex identification, labeling, and uptake in H9C2 cells

**Fig. 1:**
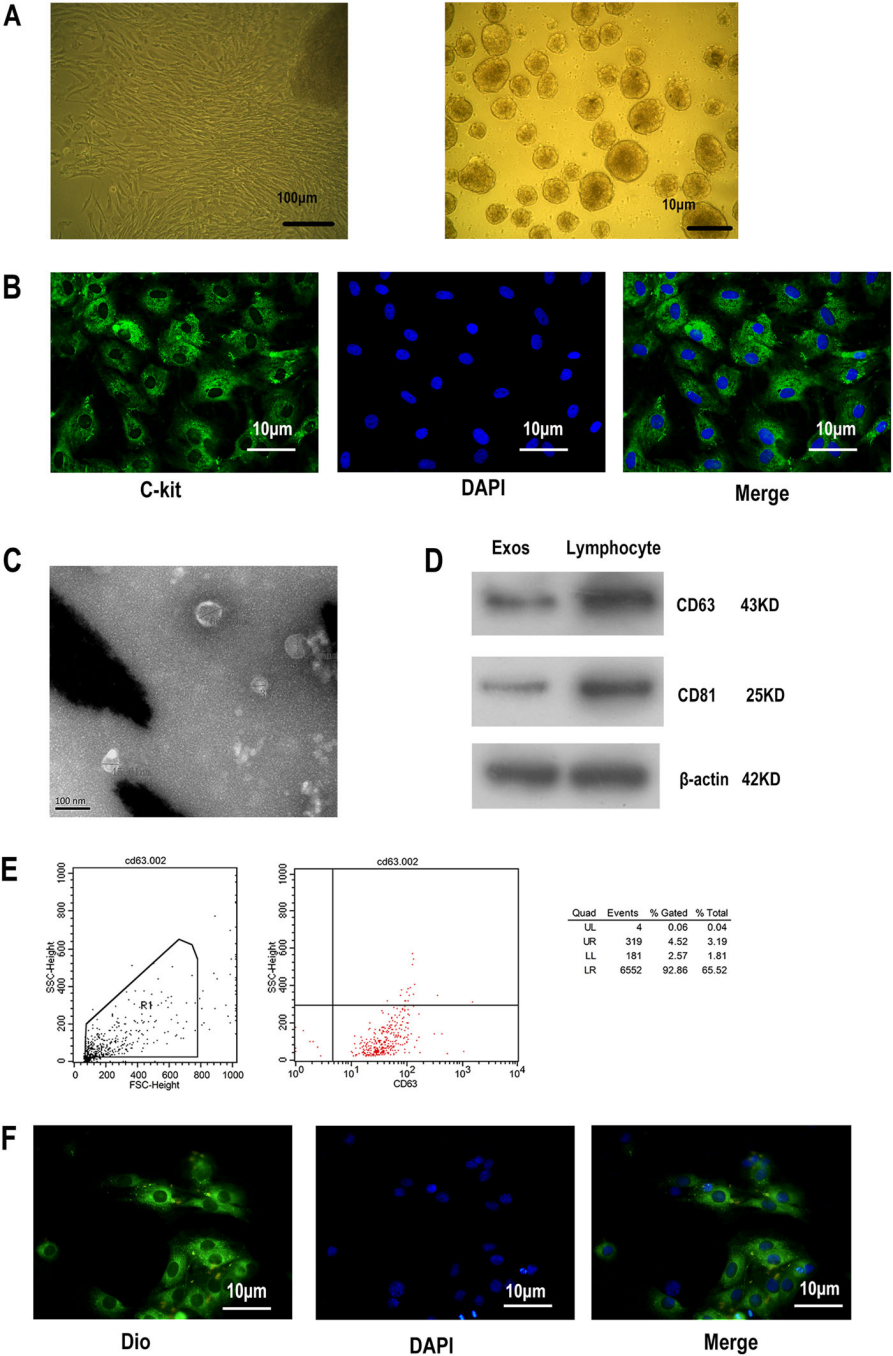
Identification and uptake of CPC-derived exosomes. **a** CPCs were isolated from Sprague-Dawley rat heart tissue (magnification, ×200) Scale bar: 100 and 10 μm. **b** CPCs were identified via c-kit make. **c** Exosomes were prepared and purified using the ExoQuick-TCTM method, and observed using TEM, showing small vesicles of 32.61 + 3.67 nm in diameter. The arrows indicate exosomes. Scale bar: 100 nm. **d** Marker proteins CD63 and CD81 of exosomes were examined via western blot analysis, using rats lymphocyte lysate as controls. **e** Marker proteins CD63 of exosomes were examined by FCM. **f** Uptake of DioC18(3)-labeled exosomes by H9C2 in vitro (magnification, ×400). Green dots indicate CPC-derived exosomes incorporated in H9C2 cells. CPCs, cardiac progenitor cells. Scale bar: 10 μm

To extract the exosomes, the CPCs were isolated and cultured from the SD rats heart tissue firstly (Fig. 1a), then identified by c-kit makers (Fig. 1b). The isolated CPCs-Ex were generated as described above and were visualized and detected using TEM and FCM. As we shown, exosomes were 30–100 nm in diameter on average (Fig. 1c). Compared with the lymphocyte lysates, samples have been demonstrated to contain a large number of exosomes, revealed that the tetraspanin molecule CD63 and CD81 were abundant in CPCs-Ex (Fig. 1d). And FCM also showed that CPCs-Ex containe a lot of CD63 (Fig. 1e). Furthermore, we labeled exosomes with DioC18(3), a fluorescent cell linker compound that is incorporated into the cell membrane via selective partitioning. After incubating the labeled CPCs-Ex with H9C2 cells, we observed a green fluorescence in the cytoplasm in almost every H9C2 (Fig. 1f).

### CPCs-Ex reduce the CVB3-induced H9C2 cell apoptosis via depressing the expression of VP1, blocking CVB3-induced Bim and Bax activation and cleavage of caspase-9 and caspase-3 and promoting the expression of BcL-2 in vitro

**Fig. 2:**
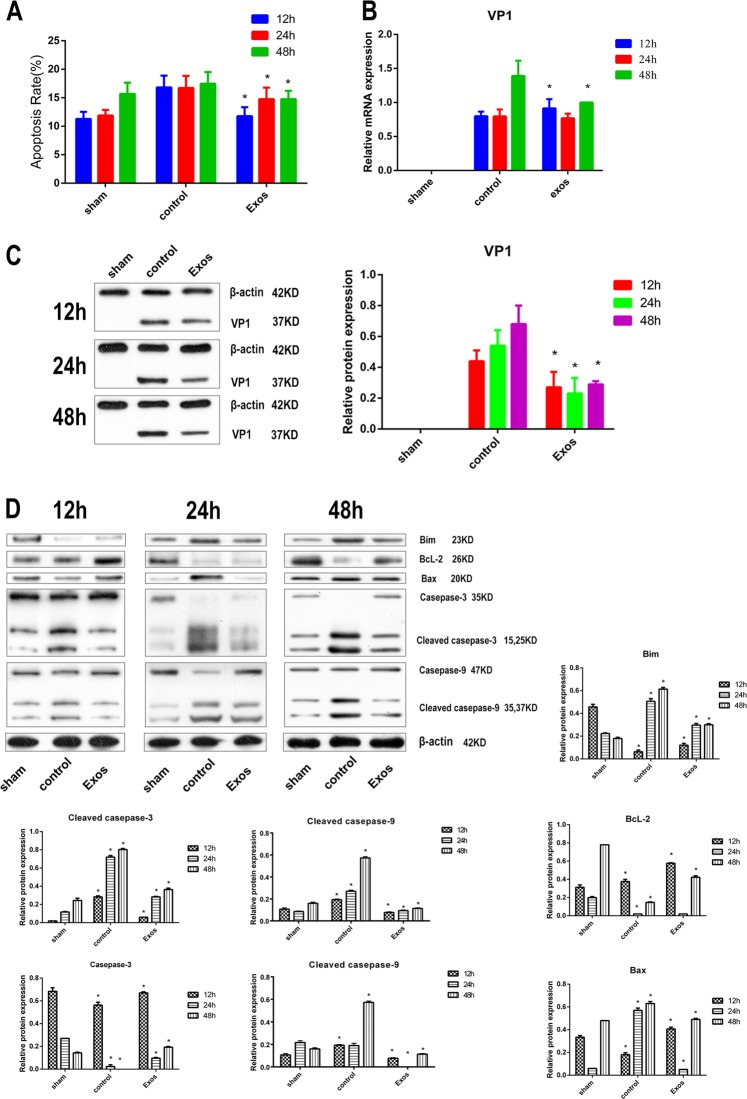
CPCs-Ex reduce the CVB3-induced H9C2 cell apoptosis in vitro. H9C2 cells were collected at 12, 24, and 48 h after infected with CVB3. **a** Depressed apoptosis rate of H9C2 cells in Exos groups compared with control (mean ± SEM, *n* = 3, one-way ANOVA). **b**, **c** Depressed mRNA and protein level of VP1 in Exos groups compared with control (mean ± SEM, *n* = 3, one-way ANOVA). **d** CPC-EX blocked CVB3-induced Bim and Bax activation and cleavage of caspase-9 and caspase-3 and promoting the expression of BcL-2 (mean ± SEM, *n* = 3, one-way ANOVA). *P *<* 0.05 vs. Control

To illustrate the relation between CPCs-Ex and the CVB3-induced apoptosis, we established the CVB3 infection cell modal, moreover, CPCs-Ex was added with 200 ng/mL at 1 h later after the CVB3 infection. After 24 and 48 h p.i., the expression of CVB3, Bim, Bcl-2, caspase-3, caspase-9 using real-time fluorescence quantitative PCR (RT-PCR) and western blot (WB) analysis and apoptosis rate was detected by flow cytometry (FCM). We found that compared with the control, CVB3-induced apoptosis was inhibited by the CPCs-Ex at 12, 24, and 48 h p.i. (Fig. 2a & supplementary file 1*, P* *<* 0.05), following the abrogated VP1 expression (Fig. 2b, c, *P* *<* 0.05). As the anti-apoptosis factor, the activation of Bcl-2 induced by CVB3 infection was further activated, while the expressions of the pro-apoptosis factors Bim and Bax were inhibited. It was also noteworthy that like the effect of caspase-9 and caspase-3, the provoked cleavage of them was prevented during the infection course (Fig. 2d, *P* *<* 0.05).

### CPCs-Ex stimulate the Akt/mTOR signaling pathway and its phosphorylation suppressed by CVB3 infection

**Fig. 3:**
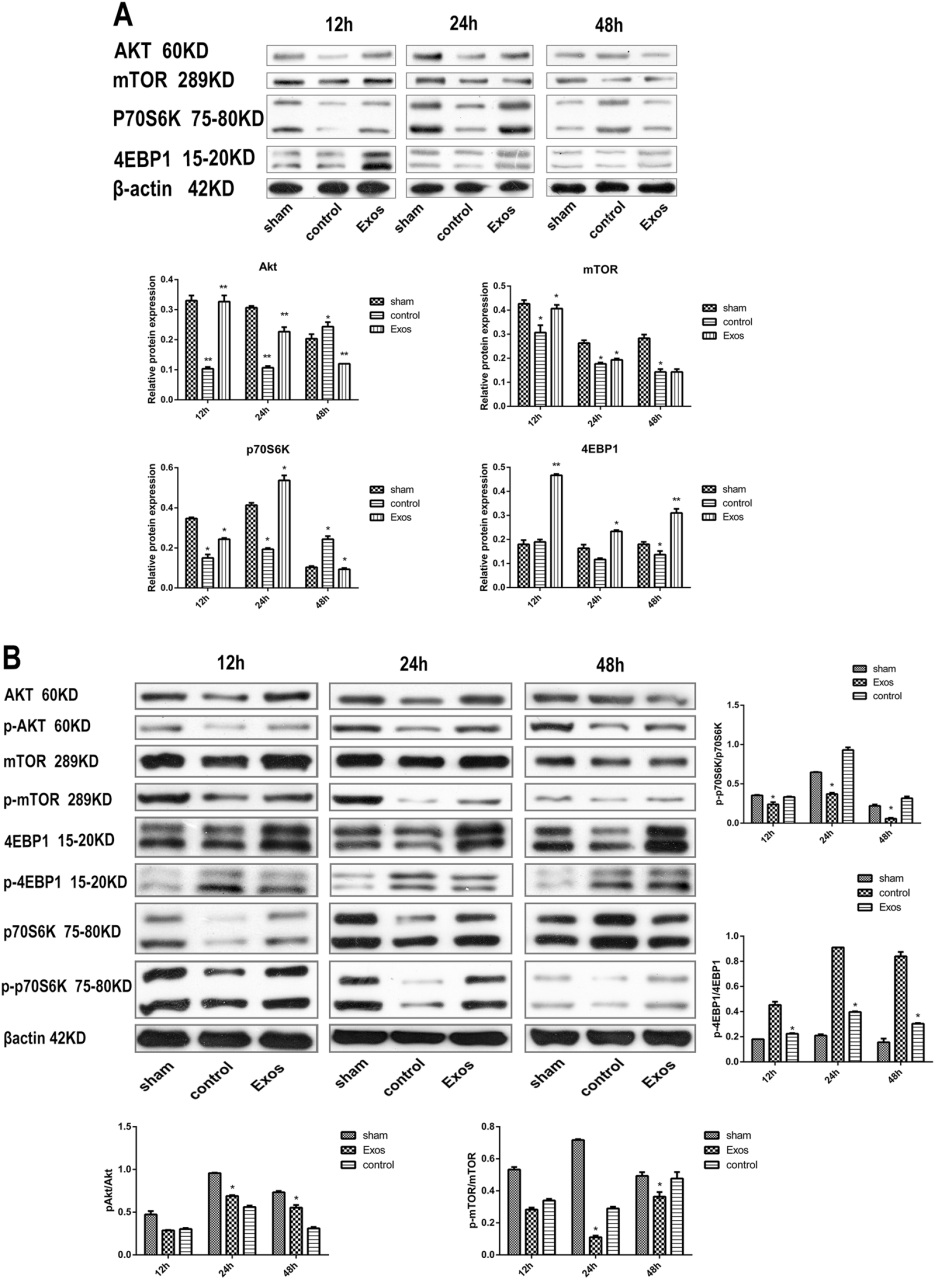
CPCs-Ex stimulate the Akt/mTOR signaling pathway and its phosphorylation. H9C2 cells were collected at 12, 24, and 48 h after infected with CVB3. **a** Protein levels of Akt, mTOR, p70S6K, and 4EBP1 were suppressed by CVB3 in control groups compared with sham and promoted by CPC-EX in Exo groups compared with control groups at 12 and 24 h (mean ± SEM, *n* = 3, one-way ANOVA). **b** Phosphorylation levels of Akt, mTOR, and p70S6K were suppressed by CVB3 infection and promoted by CPC-EX treatment (mean ± SEM, *n* = 3, one-way ANOVA). *P *<* 0.05 vs. Control

Our previous studies have shown that the Akt/mTOR signaling pathway plays an important role in anti-apoptosis and regulation of cellular transcription by activating p70S6K and 4EBP1. To further elucidate whether the mTOR signaling pathway is involved in the anti-apoptotic effect of CPCs-Ex, we measured the expression levels of related proteins and their phosphorylation levels. After adding CPCs-Ex to the H9C2 cells, protein expression of Akt, mTOR, p70S6K, and 4EBP1 was increased at 12 and 24 h, but at 48 h, protein expression of Akt and p70S6K was decreased, 4EBP1 was increased, and mTOR had no significant changes (Fig. 3a, *P* *<* 0.05). In the process of infection, the phosphorylation levels of mTOR and p70S6K were suppressed by CVB3 infection and repromoted by CPCs-Ex, but that change of 4EBP1 was on the contrary (Fig. 3b, *P* < 0.05).

### Injection of CPCs-Ex in CVB3-induced myocarditis rats can attenuate cardiomyocyte apoptosis, repair the cardiomyocyte function and reduce CVB3 replication by regulating the Akt/mTOR pathway

**Fig. 4:**
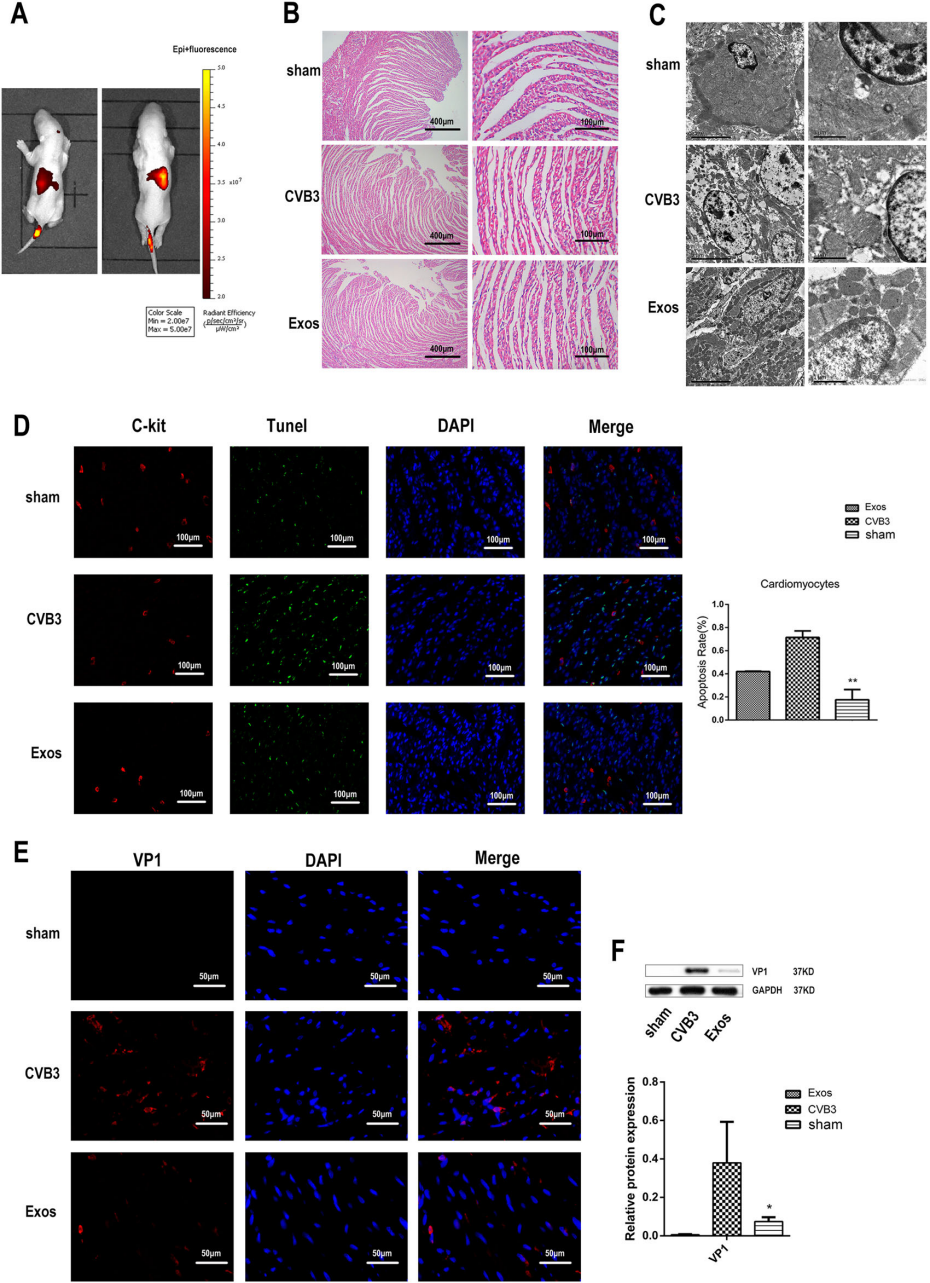
Injection of CPC-EX in CVB3-induced myocarditis rats attenuated cardiomyocyte apoptosis and reduce CVB3 replication by regulating the Akt/mTOR pathway. **a** Representative bioluminescent pictures of myocarditis rats undergone tail vein injection with CPC-EX.The signal intensity of DIR-labeled CPCs-Ex in rats represented the amount of CPCs-Ex. **b** HE staining slides of hearts dissected from myocarditis rats from different groups shown that cardiomyocytes were swollen, deformed, and disordered in CVB3 groups. Scale bar: 400 and 100 μm. **c** TEM observation of cardiomyocytes shown nuclear fragmentation, nuclear shrinkage, and other cytopathic effects in the CVB3 group. Scale bar: 5 and 1 μm. **d** TUNEL analysis of hearts tissues from different groups (mean ± SEM, *n* = 3, one-way ANOVA). Scale bar: 100 μm. **e**, **f** Immunofluorescence and western blot analysis of VP1 of hearts tissues from different groups (mean ± SEM, *n* = 3, one-way ANOVA). Scale bar: 50 μm. *P *<* 0.05 and **P < 0.01 vs. Control

To further explore the anti-apoptotic effect of CPCs-Ex in vivo, we established a rat model of CVB3-induced myocarditis. The rats were injected with CPCs-Ex through the tail vein and imaged under IVIS Lumina III In Vivo Imaging System (Fig. 4a). Forty-eight hours later, the rats were sacrificed and dissected for heart tissues, then analized by HE staining and TEM (Fig. 4b, c). The apoptosis rate of cardiomyocytes and the expression of VP1, apoptosis-related proteins and Akt/mTOR pathway-related proteins of each group were measured. After injection of CPCS-EX, the apoptotic rate of rat cardiomyocytes was significantly lower than that of the control group (Fig. 4d, *P* < 0.05). The expression of VP1 in cardiomyocytes of EXO group was significantly lower than that of the control group by western blot and immunofluorescence (Fig. 4e, f). In the meantime, through detecting the myocardial enzyme CK-MB and cTnI, we found that CPCs-Ex drop the elevated enzyme CK-MB and cTnI, improve the myocardial function (supplementary file 2, *P* < 0.05). Furthermore, the injection of CPCs-Ex promoted the expression of BcL-2, depressed that of Bim, and also inhibited the cleavage of caspase-3 and caspase-9 (supplementary file 3, *P* < 0.05). Different from the results of H9C2 cell line, the protein expression levels of Akt, mTOR, p70S6K, and 4EBP1 in the rats had not changed much after the injection of CPCs-Ex. The results of phosphorylation were consistent with the cell experiments, which is the phosphorylation of Akt, mTOR, and p70S6K was enhanced and that of 4EBP1 was inhibited compared with the control groups (supplementary file 4, *P* < 0.05).

### CPCs-Ex could decrease CVB3-induced apoptosis and suppress CVB3 expression with MK-2206 or rapamycin pretreatment by decreasing Bim/Bax expression and cleavage of caspase-3 in H9C2 cells

**Fig. 5:**
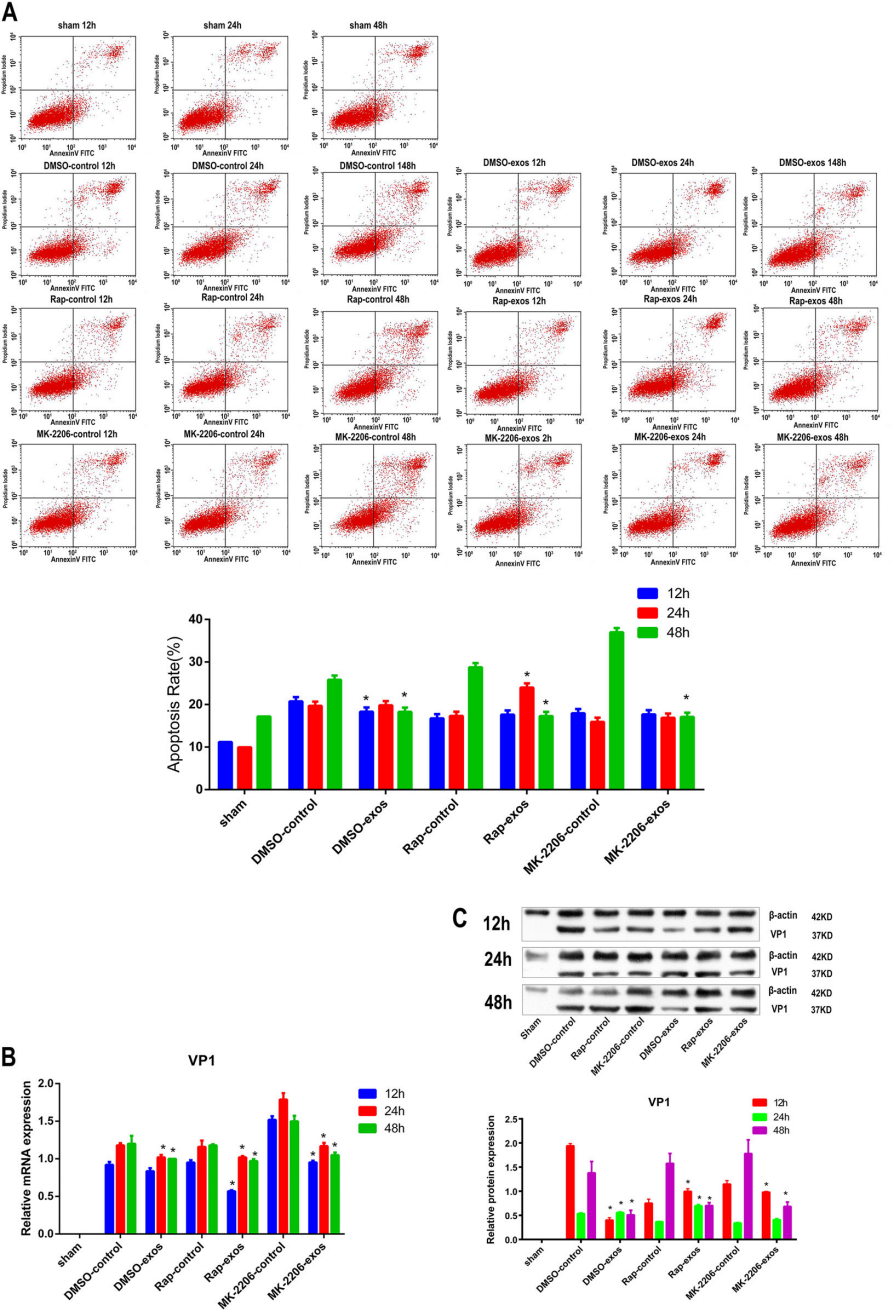
CPCs-Ex could decrease CVB3-induced apoptosis and suppress CVB3 expression with MK-2206 or rapamycin pretreatment. The H9C2 cells were pretreated with Rapamycin and MK-2206 for 30 min, then co-cultured with 200 ng/mL CPC-Ex (or PBS for control groups) for 24 h, followed by infection with CVB3. H9C2 cells were collected at 12, 24, and 48 h.p.i. and subjected to cell apoptosis, western blot, and RT-PCR analysis. **a** Apoptosis rate of H9C2 cells in different groups analyzed by FCM (mean ± SEM, *n* = 3, one-way ANOVA). **b**, **c** VP1 expression of H9C2 cells in different groups analyzed by RT-PCR and western blot (mean ± SEM, *n* = 3, one-way ANOVA). *P *<* 0.05 and **P < 0.01 vs. Control

In this study, we used 10 nmol/L Rapamycin (Rap) and 2.5 µmol/L MK-2206 to pretreat H9C2 cells, respectively, for 30 min, then cells were infected with CVB3 for 1 h, and finally 200 ng/mL of CPCs-Ex were added to cells. After cultivating for 48 h, cells were measured for apoptosis rate, CVB3 expression and protein levels of pro-apoptosis factors. We found that CPCs-Ex could significantly decrease cell apoptosis with Rap or MK-2206 pretreatment compared with control (Fig. 5a, *P* < 0.05). The results of RT-PCR showed that expression of CVB3 mRNA decreased after CPCs-Ex treatment in Rap or MK-2206 groups (Fig. 5b, *P* < 0.05). Compared with control, the VP1 expressions were suppressed by CPCs-EX in MK-2206 groups, but promoted by CPC-EX in Rap groups at 12 and 24 h p.i. (Fig. 5c, *P* < 0.05). In addition, CPCs-Ex decreased Bim, BcL-2, and Bax expression and cleavage of caspase-3 but increased cleavage of caspase-9 in Rap or MK-2206 groups compared with control (supplementary file 5, *P* *<* 0.05).

### CPCs-Ex suppressed the phosphorylation of 4EBP1 and p70S6K with MK-2206 pretreatment but strengthened that with the pretreatment of Rapamycin

To further explore the mechanism under the change of CVB3 expression with MK or Rap pretreatment, we then measured the phosphorylation of Akt, mTOR, 4EBP1, and p70S6K in H9C2 cells. After the treatment of CPCs-Ex, the phosphorylation of Akt, 4EBP1, and p70S6K was strengthened in Rap groups compared with control (*P* < 0.05). And the phosphorylation levels of mTOR, 4EBP1, and p70S6K were decreased at 48 h p.i. by CPCs-Ex in MK-2206 groups compared with controls (supplementary file 6, *P* < 0.05).

### CPCs-Ex promoted CVB3-induced apoptosis in Akt1 Akt2 4EBP1 and p70S6K overexpression groups by promoting VP1 expression and cleavage of caspase-3 at 48 h p.i.

**Fig. 6:**
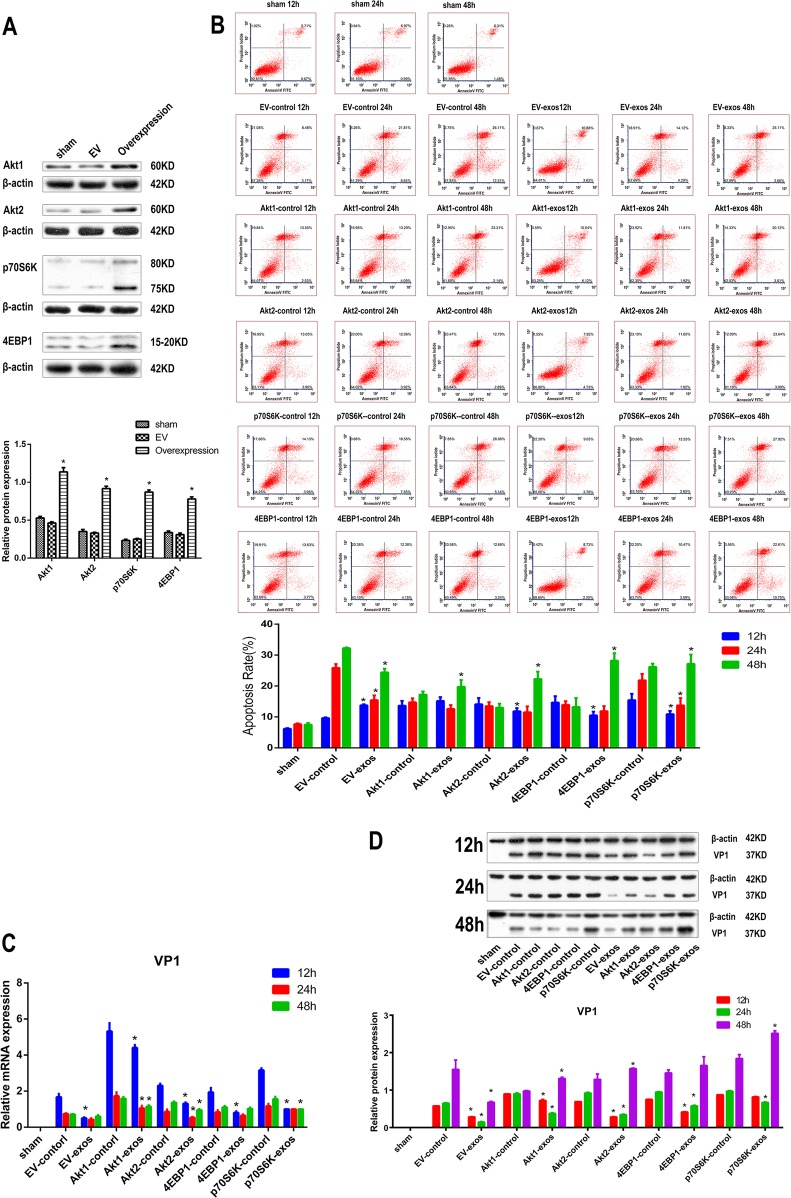
CPCs-Ex promoted CVB3-induced apoptosis and VP1 expression in Akt1, Akt2, 4EBP1, and p70S6K overexpression groups **a** Establishment of stable cell lines overexpressing 4EBP1, p70S6K, Akt1, and Akt2, respectively. The expressions of Akt1, Akt2, 4EBP1, and p70S6K were determined by western blot analysis. **b** Apoptosis rate of H9C2 cells in different groups analyzed by FCM (mean ± SEM, *n* = 3, one-way ANOVA). **c**, **d** VP1 expression of H9C2 cells in different groups analyzed by RT-PCR and western blot (mean ± SEM, *n* = 3, one-way ANOVA). *P *<* 0.05 and **P < 0.01 vs. Control

**Fig. 7:**
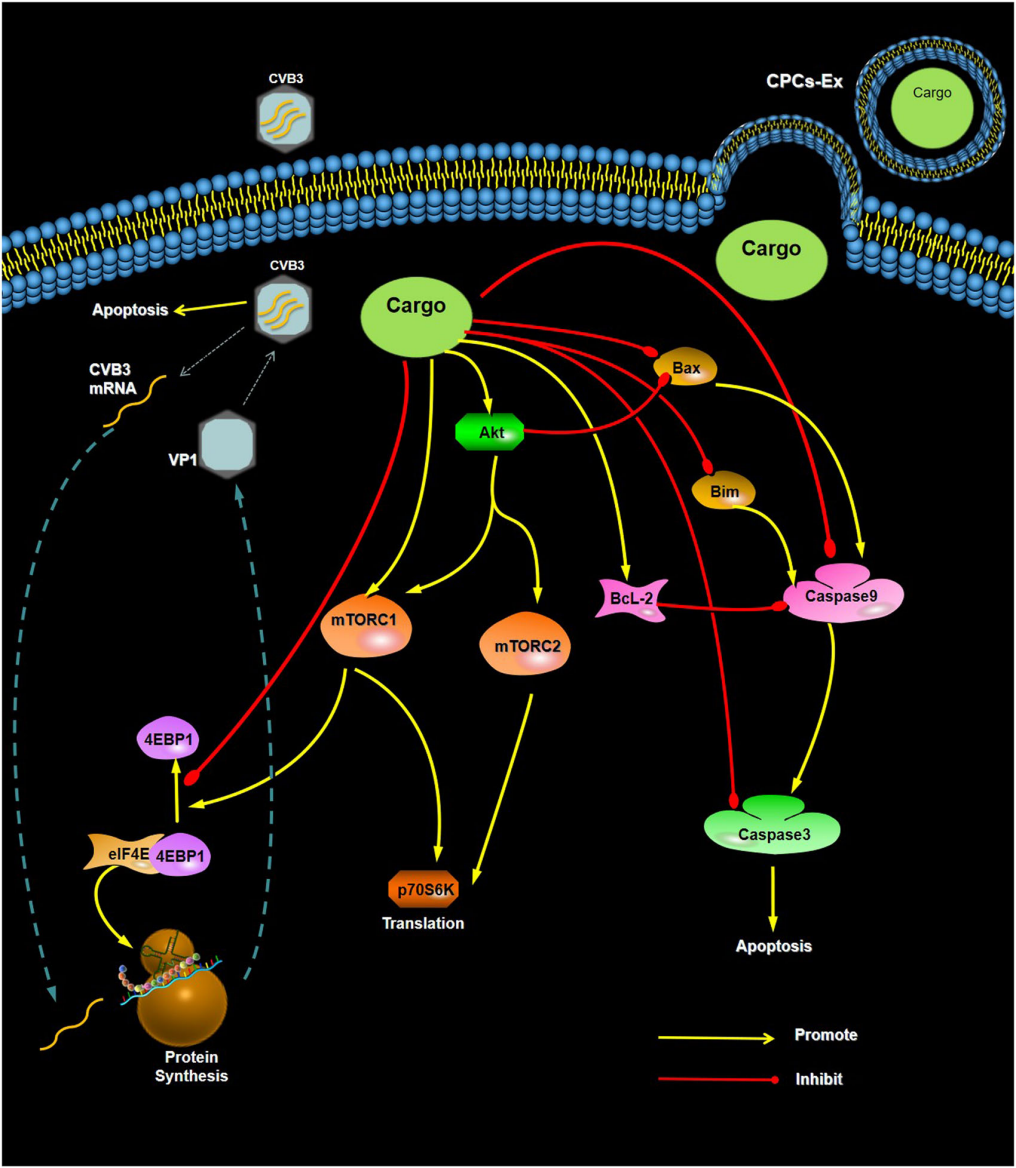
CPCs-Ex attenuate CVB3-induced apoptosis via suppressing CVB3 replication and BcL-2/caspase families. This example shown CPCs-Ex could suppress CVB3-induced apoptosis in several ways. First, CPCs-Ex stimulated Akt and downstream factors to suppress pro-apoptotic factors. Second, CPCs-Ex derectly blocked BcL-2 and caspase families. Third, CPCs-Ex could suppress CVB3 replication by inhibiting the Phosphorylation of 4EBP1

As we described above, CPCs-Ex seems utilized the mTOR signaling pathway to mediate the CVB3-induced apoptosis. To further investigate the relationship between mTOR signaling pathway and CPCs-Ex during the process of apoptosis, we established the eukaryotic expression plasmids overexpressed the Ratus Akt1, Akt2, 4EBP1, or p70S6K, which were stable transfected to H9C2 cells. The expressions of Akt1, Akt2, 4EBP, and p70S6K were determined by western blot analysis (Fig. 6a). After infected with CVB3 for 1 h, CPCs-Ex were added with 200 ng/mL. Unexpectedly, CPCs-Ex decreased apoptotic rate at 12 h but increase that at 48 h p.i. in the four overexpression groups compared with controls (Fig. 6b, *P* < 0.05). RT-PCR shown that the expression of CVB3 mRNA decreased in the overexpression groups with CPCs-Ex treatment compared with controls p.i. (Fig. 7c, *P* < 0.05). And western blot assays showed VP1 expression were decreased at 12 and 24 h p.i. but increased significantly at 48 h p.i. in Akt2, 4EBP1, and p70S6K overexpression groups after CPCs-Ex treatment (Fig. 6d, *P* < 0.05). Besides, Bax and Bim expression was both decreased in the four overexpression groups by CPCs-Ex at 24 and 48 h p.i. But at the late stage of infection, the cleavage of caspase-3 was promoted and the cleavage of caspase-9 was repressed in Akt2, 4EBP1, and p70S6K overexpression groups (supplementary file 7, *P* < 0.05).

### CPCs-Ex promoted phosphorylation of Akt mTOR and P70S6K at early stage p.i. and stimulated phosphorylation of 4EBP1 at late stage of infection in overexpression groups

We then measured the phosphorylation levels of Akt, mTOR, 4EBP1, and P70S5K in the overexpression groups. After the treatment of CPCs-Ex, the phosphorylation levels of Akt, mTOR, and p70S6K were increased at 12 and 24 h in almost all the four overexpression groups. However, the phosphorylation of 4EBP1 was suppressed at early stage but stimulated at 48 h p.i. in Akt1, Akt2, and P70S6K groups (supplementary file 8, *P* < 0.05).

## Discussion

As a major cause of sudden cardiac death in youth, VMC still lack specific and effective treatment^25^. Years of research have confirmed that the role of CPCs in the repair of the heart is mainly through the paracrine, rather than directly differentiate into cardiomyocytes^6,26^^.^ As an important component of paracrine secretion, exosomes participate in processes such as immune response, antigen presentation, cell migration, cell differentiation and tumor invasion^9,27^. Cardiac-, plasma- and stem-cell-derived exosomes were reported to be cardioprotective by numerous studies, among which CPCs-Ex was the most promising one^28,29^. A recent research has showed that the efficacy of rat-derived CPCs-Ex stimulates after hypoxia^30^. Just as our results shown, the CVB3-induced increase of CK-MB and cTnI in rats were repaired after the injection of CPCs-Ex, which directly demonstrated the cardioprotection of CPCs-Ex. Besides, the miRNA content in the exosomes also changed with hypoxia, implying that there are some possible compensatory pathways exist in the CPCs to alter exosome secretion on the basis of microenvironmental clues^31^. Arslan et al.^32^ have reported that exosomes derived from stem cells improve myocardial viability after injury and alleviate adverse remodeling of the damaged heart due to the activation of AKT pathway and the reduction in oxidative stress in a myocardial infarction (MI) model in vivo. It is precisely because this lack of mechanism and effective treatment in the VMC, based on our previous data, our present study devoted to illuminate the relationship between CPCs-Ex and the Akt/mTOR in CVB3-induced apoptosis, proving that CPCs-Ex could suppress the CVB3-induced apoptosis and replication in H9C2 cells and myocarditis rat model, which indicating that CPCs-Ex could be a novel and significant therapeutic strategy for VMC.

The dysregulation of the apoptotic pathway is an important pathological process of CVB3-induced VMC^33^, during which BcL-2 family and the caspase family play important regulatory roles in the transduction pathway, especially the mitochondrial pathway^34^. In this study, we measured the expression of BcL-2 and caspase families, to reflect the change of apoptosis pathways under CVB3 infection and CPCs-Ex treatment. In present research, the data implied that CPCs-Ex could alleviate the apoptosis induced by CVB3 both in vivo and in vitro. Further, we found that the expressions of Bim and Bax in CPCs-Ex groups were lower than those in CVB3 groups. But just the opposite, as an anti-apoptosis protein, the expression of Bcl-2 in CPCs-Ex was increased. In the meantime, compared with the CVB3 group, the cleaved caspase-9 and caspase-3 were inhibited in the CPCs-Ex group. Bim and Bax are well known to be the pro-apoptosis, which can promote the major core of intrinsic apoptosis signal pathway. Caspase family is another common mediator of apoptosis, of which caspase-9 contributes to the apoptosis origination while caspase-3 participates in the execution. It is the common concept that the activated Bax contributes to form channels on the mitochondrial membrane leading to cytochrome C release^35,36^, which can lead to caspase-9 self-cleave, then further processes other caspase members, such as initiating a caspase cascade including caspase-3. Those two families are interacted. Accordingly, our data indicate that CPCs-Ex could reduce the CVB3-induced apoptosis via inhibiting the virus replication, activating Bcl-2 expression and suppressing the activation of Bim, Bax and the self-cleavage of caspase-9 and caspase-3 induced by CVB3 infection simultaneously.

Several studies have shown that stem-cell-derived exosomes activate the Akt/mTOR pathway to counteract apoptosis or promote cell growth during the repair of cell damage^37–39^. And Akt/mTOR pathway were also reported to play a pivotal role in CBV3 replication and CVB3-induced apoptosis by our precious work^24,40^. Therefore, we further detected the expression of the Akt/mTOR pathway factors in the present study. We found that protein expression and phosphorylation of Akt, mTOR, and p70S6K were increased after addition of CPCs-Ex to H9C2 cells, suggesting that CPCs-Ex may activate Akt/mTOR pathway during CVB3-induced apoptosis. It is commonly held the concepts that Akt/mTOR pathway is an anti-apoptosis factor, which is also an important mediator in the angiogenesis, protein synthesis and other cell growth process. Interestingly, when CPCs-Ex were added both in cells and rats after CVB3 infection, there was an increase in the expression of 4EBP1 and decrease of its phosphorylation, in consort with the reduced VP1 expression. It had been proved that CVB3 replication can be suppressed by non-phosphorylated 4EBP1, which can lead to suppression of translation initiation of 5′TOP mRNA^41^. In an early study, mTOR/4EBP1 signaling pathway has been demonstrated to be hosted and utilized by CVB3 to promote its own replication during the pathogenesis of VMC^42^. Therefore, CPCs-Ex could inhibit CVB3 replication therefore reduce the CVB3-induced apoptosis by inhibiting the phosphorylation of 4EBP1, which might be a novel mechanism utilized by CPCs-Ex.

To further explore the relationship of Akt/mTOR and CPCs-Ex in CVB3-induced apoptosis, we used Akt/mTOR inhibitors MK-2206 and Rap, along with the construction of stable cell lines using Akt1, Akt2, 4EBP1, and p70S6K. Compared with the control, apoptosis rate in CPCs-Ex group pretreated with Rap or MK was obviously reduced followed by the suppression of VP1 expression. While in the overexpression groups, CPCs-Ex reduced the CVB3-induced apoptosis and VP1 expression at 24 h p.i., whereas, both of that were increased at 48 h p.i. In the meantime, we found that CPCs-Ex could suppressed the phosphorylation of mTOR, 4EBP1, and p70S6K after with the pretreatment of MK, but strengthened that with the pretreatment of Rap which resulting in the decreased VP1 expression in MK groups and increased VP1 expression in Rap groups. This result may also suggest that the anti-CVB3 replication effect of CPCs-Ex may be mainly mediated by Akt/4EBP1 pathway. It is well known that mTOR has two functionally distinct complexes: mTOR complex 1 (mTORC1) and mTOR complex 2 (mTORC2)^43^. When mTORC1 is activated, it then phosphorylates the 4EBP1 and p70S6K. The phosphorylation of 4EBP1 allows cap-dependent translation to proceed. Meanwhile, the activation of p70S6K enhances the translation of mRNAs, promoting the cell growth consequently^44^. When mTORC1 was inhibited by the Rap, CPCs-Ex could increase the phosphorylation of p70S6K via the surviving mTORC2 pathway. Yet, the phosphorylation of 4EBP1 was strengthened by CPCs-Ex, which resulting in the increase replication of CVB3. An early study has demonstrated that gastric cancer exosome could supress Jurkat T cells apoptosis by stimulating downstream Akt activity^45^. Like that, our data shown CPCs-Ex enhanced Akt and its downstream phosphorylation, which decreased the viral replication and reduce CVB3-induced apoptosis consequently. In our present study, apoptosis rate and VP1 expression in CPC-Ex group were both reduced at 12 h p.i. in the groups of overexpression. Whereas, apoptosis was exacerbated by CPCs-Ex at 48 h p.i. followed by the rebound of viral replication. By adding CPCs-Ex to Akt1, Akt2, 4EBP1, and p70S6K overexpressed cells, we found that CPCs-Ex could enhance the anti-apoptosis effect of Akt1, Akt2, and p70S6K overexpression by stimulate phosphorylation of Akt, mTOR, and p70S6K. Meanwhile Akt2 and p70S6K overexpression also stimulated the phosphorylation of 4EBP1, which might contribute to CVB3 replication. It is commonly held the concepts that Akt is an anti-apoptosis factor. Besides, a recent study has reported that Akt1 activation may prevent apoptosis through upregulating of the survivin^46^. Akt2 is implicated in diverse process of cardiomyocyte signaling including survival and metabolism, whose deficiency may cause retardation of cardiomyocyte development^47^, implying a pivotal role in the cardiomyocyte survival. At early stage after viral infection, CPCs-Ex reduced the viral replication and apoptosis via decreasing the phosphorylation of 4EBP1 and p70S6K. Along with the infected time, the increased effect of CPCs-Ex on p70S6K was translated to decrease, yet the phosphorylation of 4EBP1 was stimulated, which may be utilized by CVB3 to promote the replication and then reinforce the apoptosis process.

On the other hand, we detected the Bcl-2 and caspase family members when Akt/mTOR signaling pathway was inhibited or overexpressed, respectively. We found that along with the infected time, CPCs-Ex could suppress the activated cleaved caspase-3, while keeping the cleavage of caspase-9 with the Rap or MK pretreatment. In the overexpressed groups, however, the expression of cleaved caspase-3 was further increased and the cleavage of caspase-9 was repressed. As previously mentioned, during the apoptosis process, caspase-9 contributes to the apoptosis origination and caspase-3 participates in the execution. Zhang et al.^48^ have observed that the mitochondrial membrane potential collapses and the ratio of Bax/Bcl-2 in the cytoplasm increases, inducing cytochrome c release from the mitochondria to the cytoplasm, activates caspase-9/-3 and finally induces apoptosis, implying caspase-9 and caspase-3 are responsible for the activation of apoptosis. Another study has reported that bone mesenchymal stem cells (BMSCs) derived exosome could suppress the apoptosis via reducing the cleavage of caspase-3, caspase-8, and caspase-9 directly in colitis rats. Therefore, we speculate that when Akt/mTOR signaling pathway is inhibited, CPC-Ex can synergistically mitigate CVB3-induced apoptosis via a caspase-3 dependent pathway. Exosome, theoretically treated to be an inhibitor of apoptosis and accelerator of proliferation, have been demonstrated to induce the apoptosis in Jurkat T cells via inhibiting the PI3K/Akt pathway and mediating the caspase family^45^. Thus, when more substrates of Akt/mTOR pathway were provided, CPCs-Ex was turned into the accomplice of CVB3, promoting cell apoptosis via promoting the activation of cleaved caspase-3 which induced by CVB3 infected.

In conclusion, based on our data, we suggest that CPCs-Ex could mitigate the CVB3-induced apoptosis and block CVB3 replication by inhibiting the phosphorylation of 4EBP1 and suppressing pro-apoptosis factors (Fig. 7). Moreover, the synergetic anti-apoptosis effect of CPCs-Ex rely on the Akt/4EBP1 and caspase-3 dependent pathways. Our work may provide new insights into the role of exosomes in the pathogenic mechanism and treatment on VMC, yet still requires our more in-depth research.

## Supplementary information

Supplementary Figure Legends

supplementary figure 1

supplementary figure 2

supplementary figure 3

supplementary figure 4

supplementary figure 5

supplementary figure 6

supplementary figure 7

supplementary figure 8
