# Supplementary material for: Exosomes derived from cardiac progenitor cells attenuate CVB3-induced apoptosis via abrogating the proliferation of CVB3 and modulating the mTOR signaling pathways
Source: Cell Death Dis. 2019 Sep 18;10(10):691. doi: 10.1038/s41419-019-1910-9 (PMC6751166; doi:10.1038/s41419-019-1910-9)
Supplement: Supplementary file 1 — Supplementary Figure Legends [file 41419_2019_1910_MOESM1_ESM.docx]

**Supplementary Figure Legends**

**supplementary figure 1.**Depressed apoptosis rate of H9C2 cells in Exos groups compared with control.

**supplementary figure 2.** Western blot analysis of apoptotic factors of hearts tissues from different groups. (mean±SEM,n=3,one way ANOVA).**P*<0.05 and ***P*<0.01 vs. Control

**supplementary figure 3.** Western blot analysis of myocardial injury markers of hearts tissues from different groups. (mean±SEM,n=3,one way ANOVA).**P*<0.05 and ***P*<0.01 vs. Control

**supplementary figure 4.** Phosphorylation and protein levels of Akt, mTOR, p70S6K and 4EBP1 of hearts tissues from different groups.(mean±SEM,n=3,one way ANOVA).**P*<0.05 and ***P*<0.01 vs. Control

**supplementary figure 5.** western blot analysis of apoptotic factors of H9C2 cells from different groups. (mean±SEM,n=3,one way ANOVA).**P*<0.05 and ***P*<0.01 vs. Control

**supplementary figure 6.** Phosphorylation and protein levels of Akt, mTOR, p70S6K and 4EBP1 of H9C2 cells from different groups.(mean±SEM,n=3,one way ANOVA).**P*<0.05 and ***P*<0.01 vs. Control

**supplementary figure 7** western blot analysis of apoptotic factors of H9C2 cells from different groups. (mean±SEM,n=3,one way ANOVA).**P*<0.05 and ***P*<0.01 vs. Control

**supplementary figure 8** Phosphorylation and protein levels of Akt, mTOR, p70S6K and 4EBP1 of H9C2 cells from different groups.(mean±SEM,n=3,one way ANOVA).**P*<0.05 and ***P*<0.01 vs. Control
