# Supplementary figures and images for: Exosomes derived from cardiac progenitor cells attenuate CVB3-induced apoptosis via abrogating the proliferation of CVB3 and modulating the mTOR signaling pathways
Source: Cell Death Dis. 2019 Sep 18;10(10):691. doi: 10.1038/s41419-019-1910-9 (PMC6751166; doi:10.1038/s41419-019-1910-9)

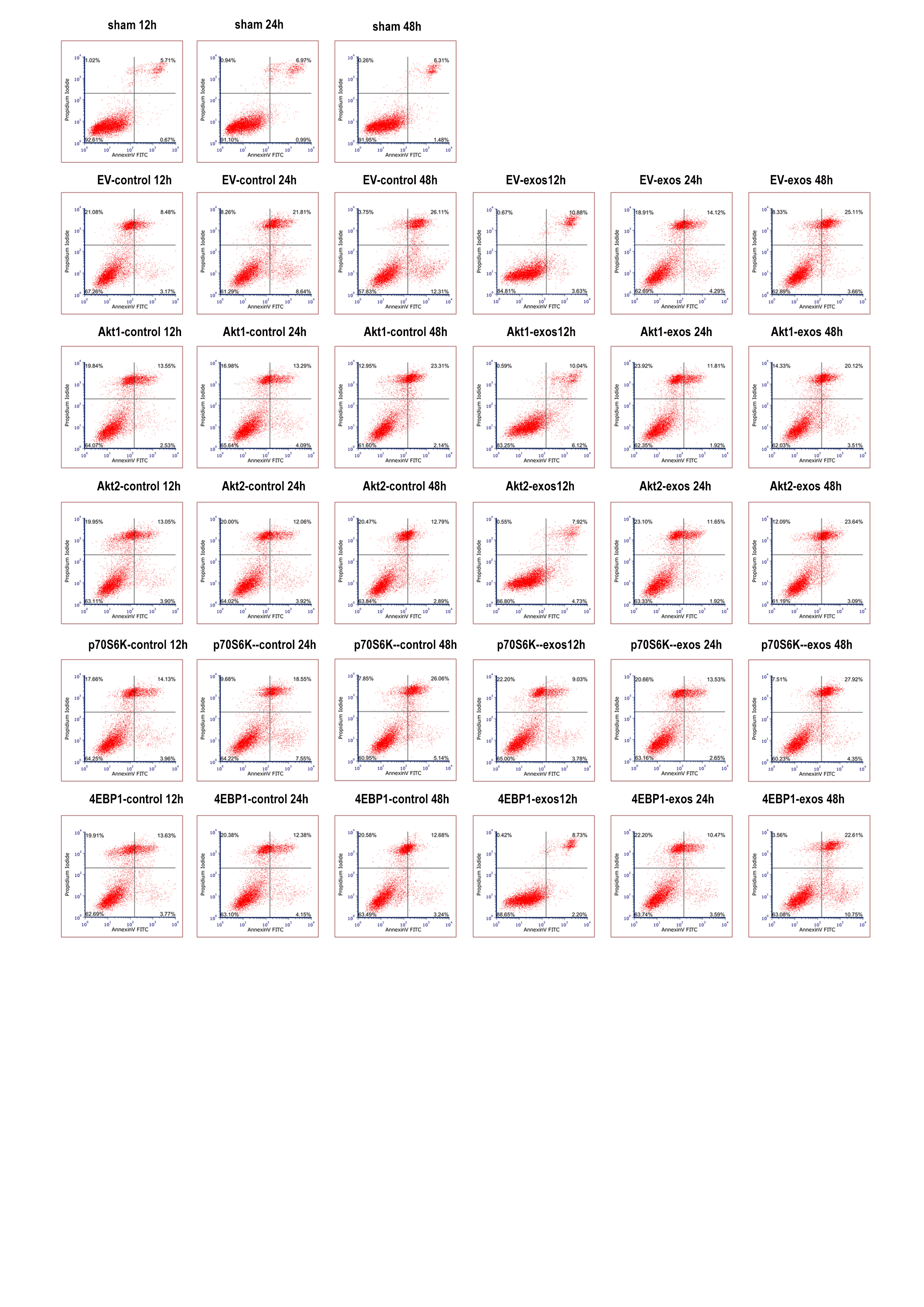

Supplement: Supplementary file 2 — supplementary figure 1 [file 41419_2019_1910_MOESM2_ESM.tif]

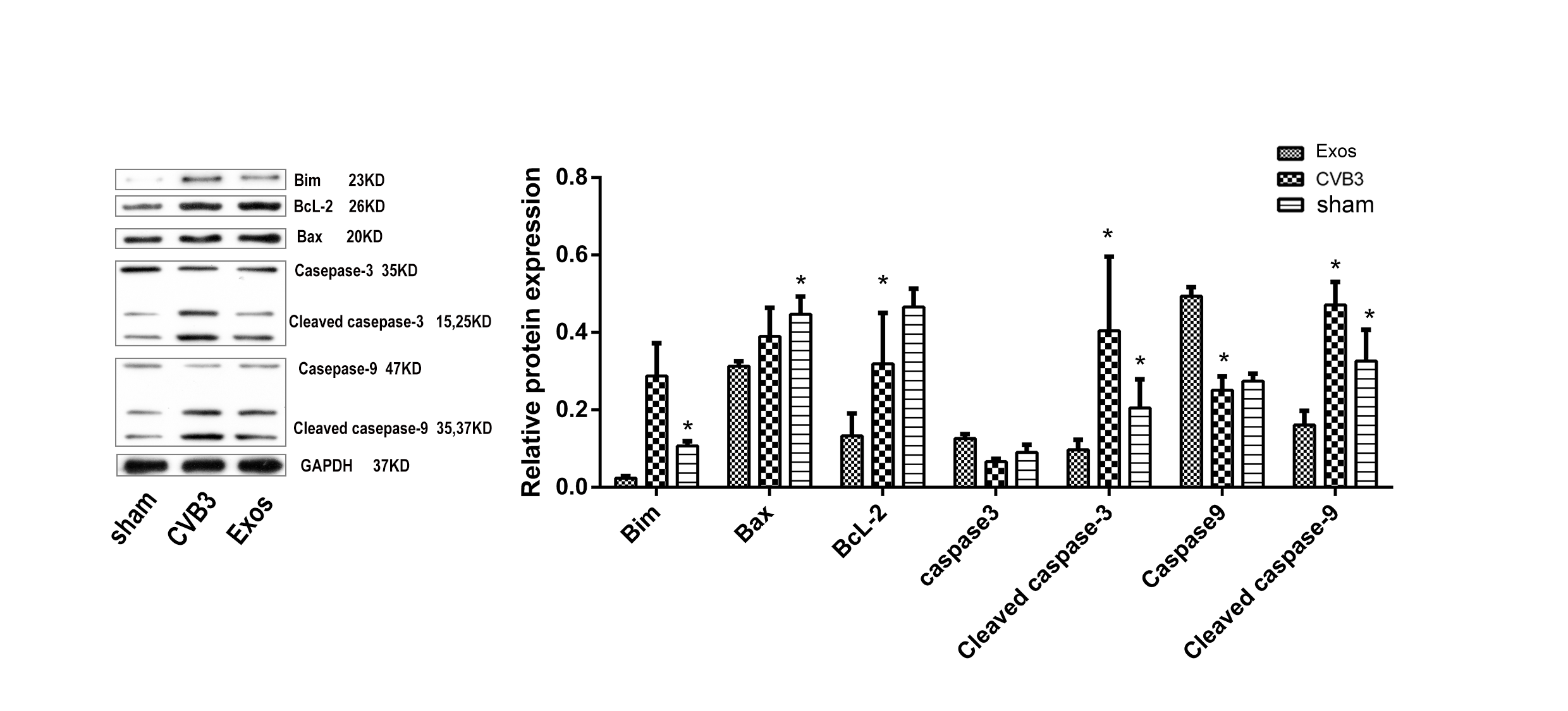

Supplement: Supplementary file 3 — supplementary figure 2 [file 41419_2019_1910_MOESM3_ESM.tif]

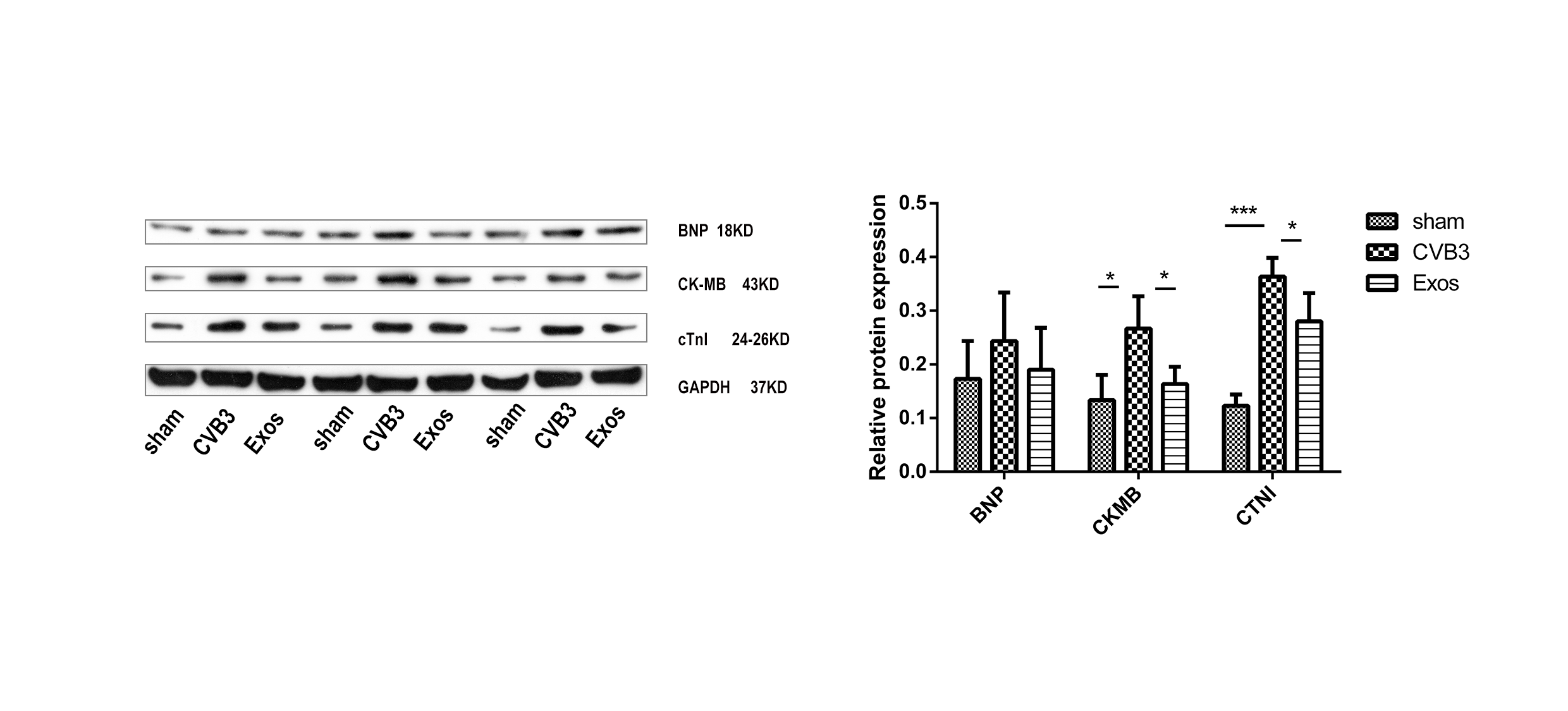

Supplement: Supplementary file 4 — supplementary figure 3 [file 41419_2019_1910_MOESM4_ESM.tif]

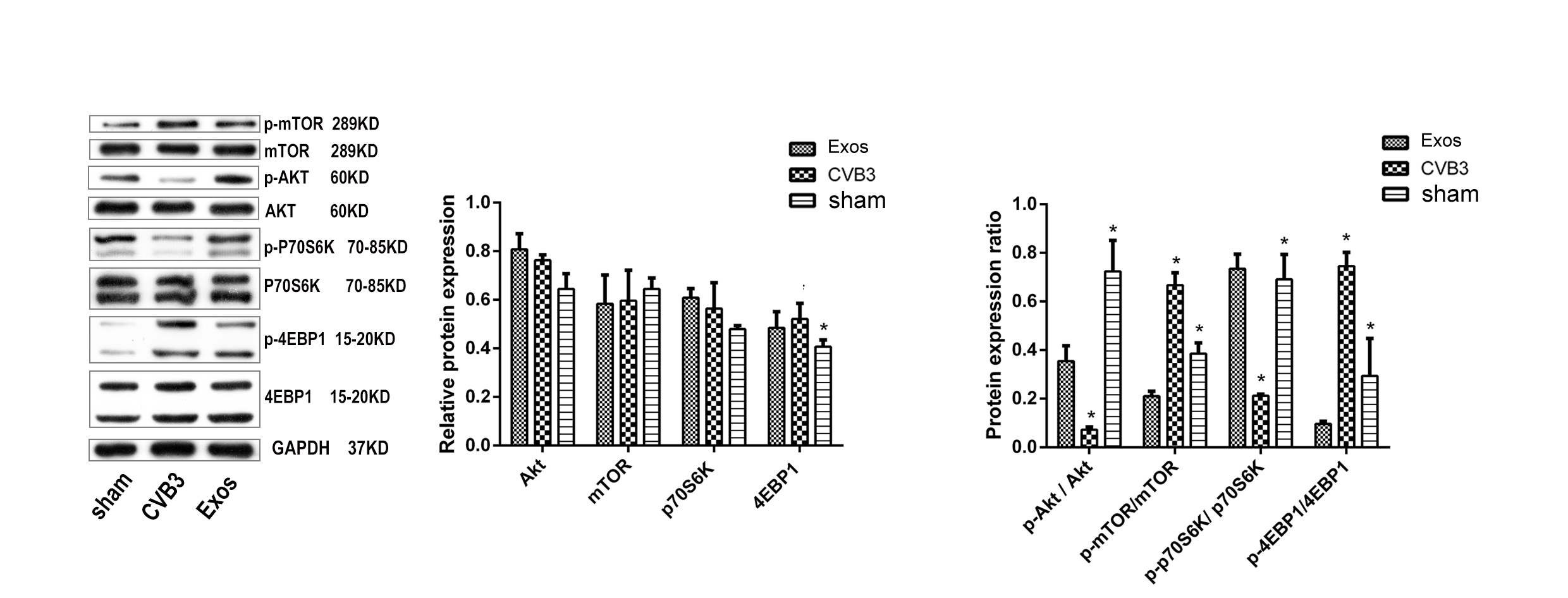

Supplement: Supplementary file 5 — supplementary figure 4 [file 41419_2019_1910_MOESM5_ESM.tif]

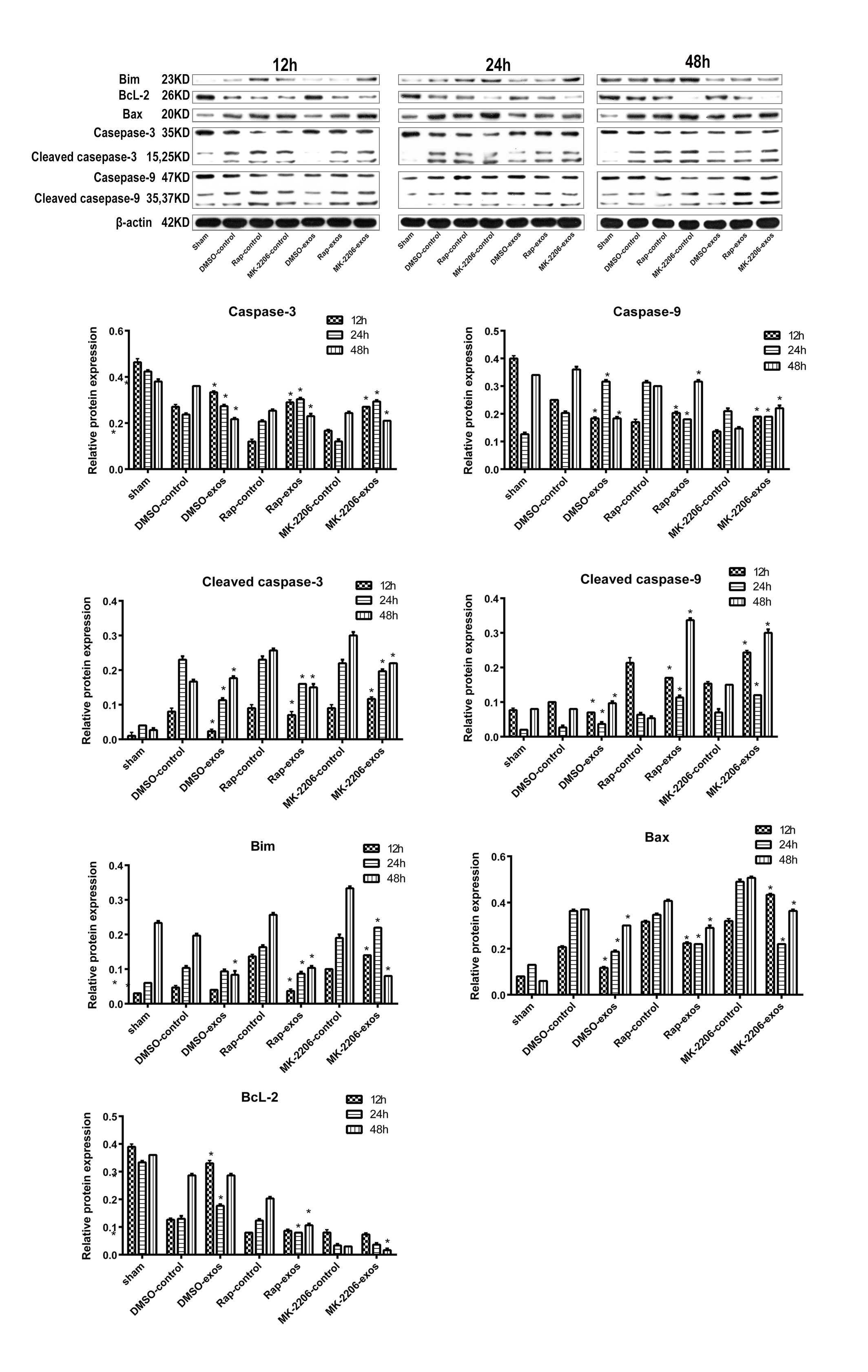

Supplement: Supplementary file 6 — supplementary figure 5 [file 41419_2019_1910_MOESM6_ESM.tif]

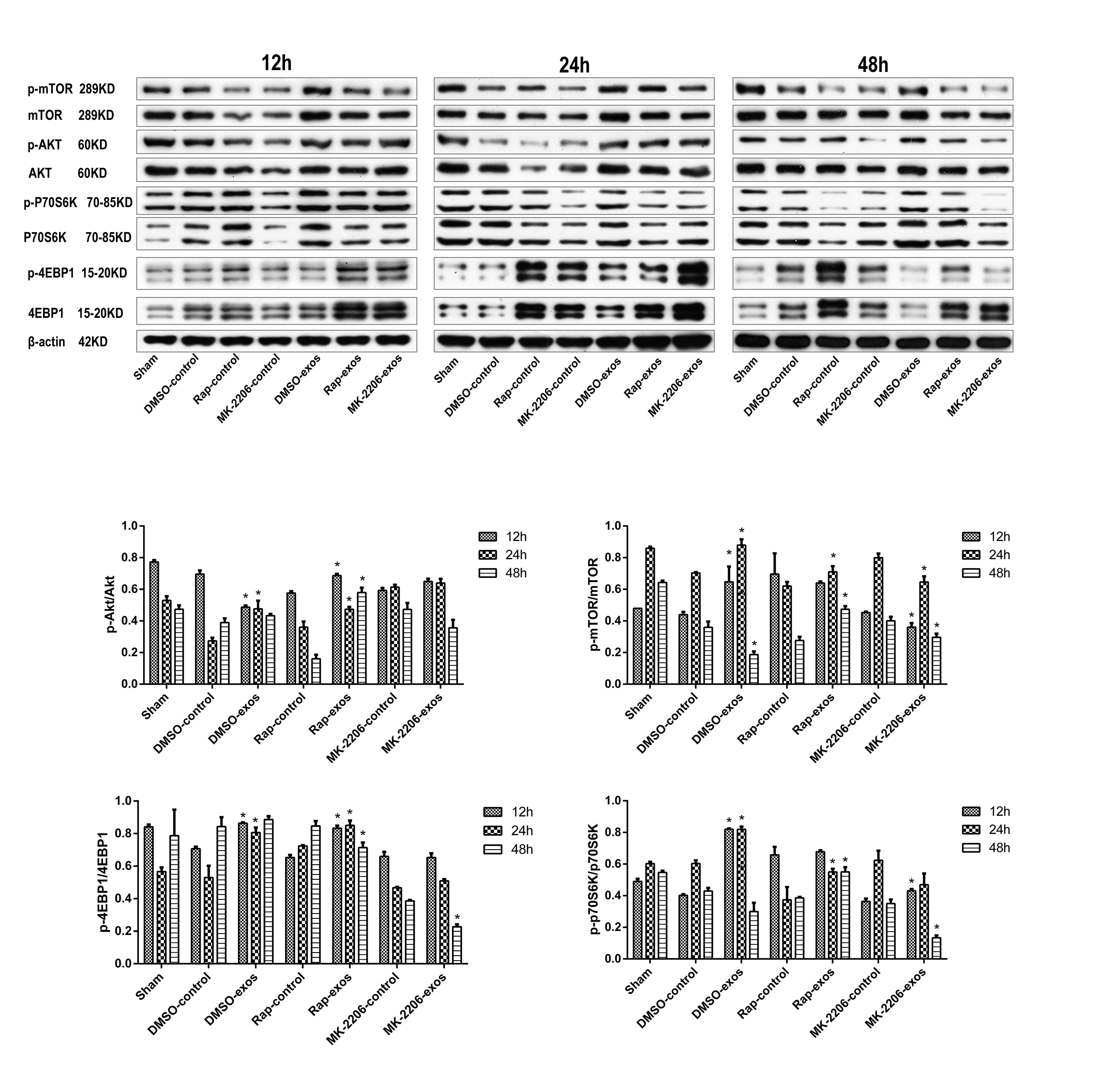

Supplement: Supplementary file 7 — supplementary figure 6 [file 41419_2019_1910_MOESM7_ESM.tif]

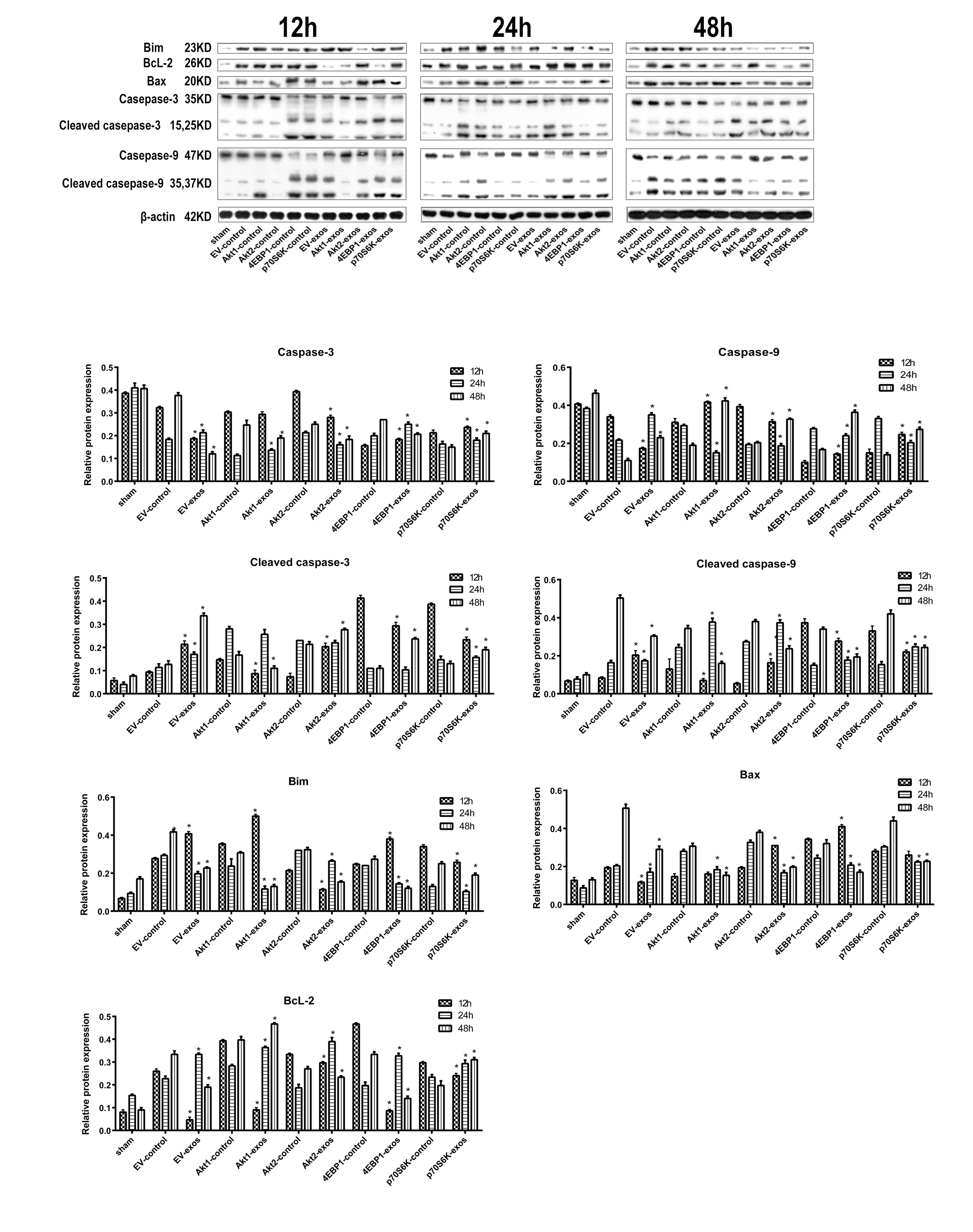

Supplement: Supplementary file 8 — supplementary figure 7 [file 41419_2019_1910_MOESM8_ESM.tif]

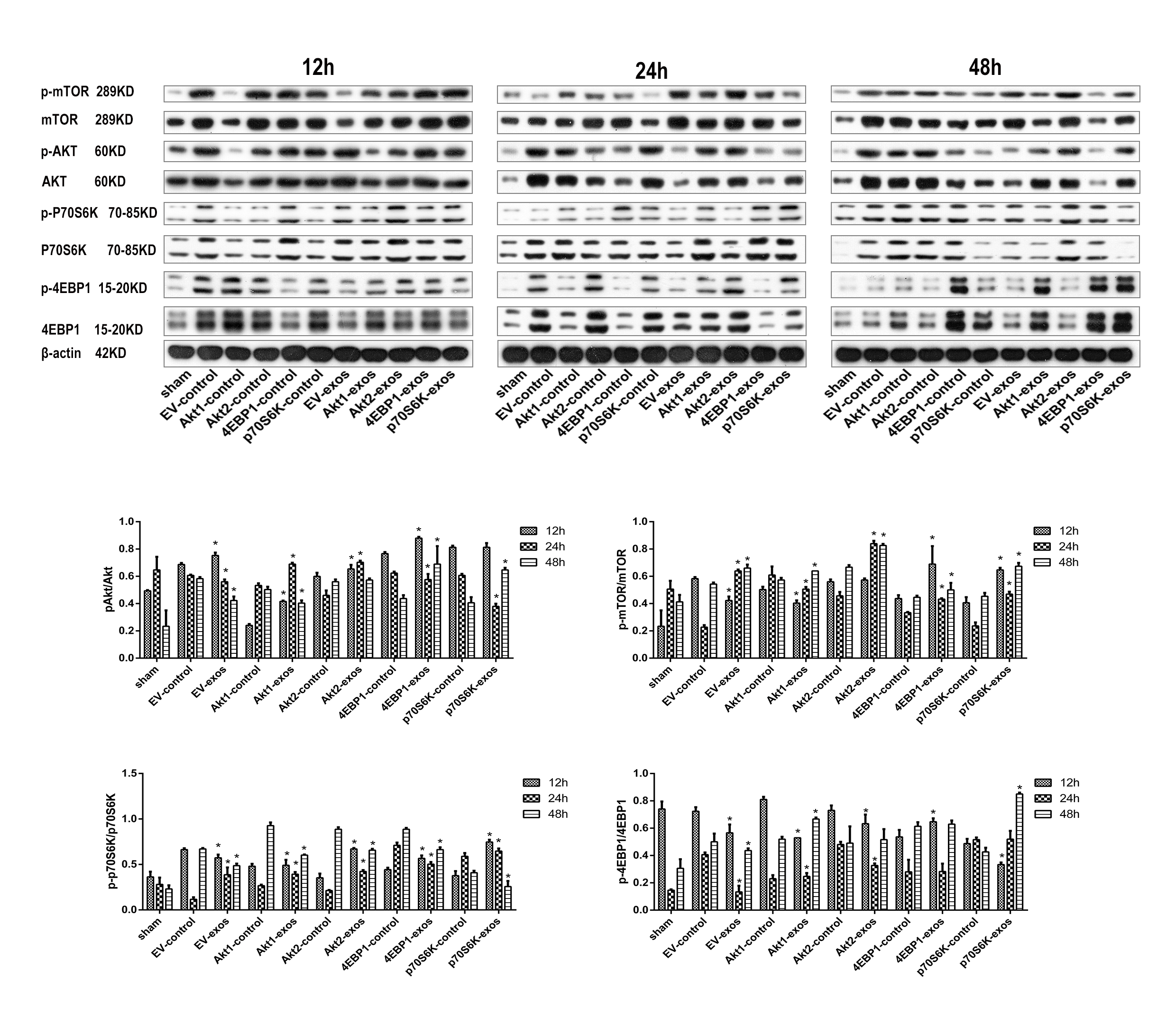

Supplement: Supplementary file 9 — supplementary figure 8 [file 41419_2019_1910_MOESM9_ESM.tif]
